# Estimates of the Prevalence of Speech and Motor Speech Disorders in Youth With 22q11.2 Deletion Syndrome

**DOI:** 10.1044/2018_AJSLP-18-0037

**Published:** 2018-12-04

**Authors:** Adriane L. Baylis, Lawrence D. Shriberg

**Affiliations:** a Department of Plastic and Reconstructive Surgery, Nationwide Children's Hospital, Columbus, OH; b Department of Plastic Surgery, The Ohio State University College of Medicine, Columbus; c Intellectual & Developmental Disabilities Research Center, Waisman Center, University of Wisconsin-Madison

## Abstract

**Purpose:**

Speech sound disorders and velopharyngeal dysfunction are frequent features of 22q11.2 deletion syndrome (22q). We report the first estimate of the prevalence of motor speech disorders (MSDs) in youth with 22q.

**Method:**

Seventeen children and adolescents with 22q completed an assessment protocol that included a conversational speech sample. Data reduction included phonetic transcription, perceptual speech ratings, prosody-voice coding, and acoustic analyses. Data analyses included 3 motor speech measures and a cross-classification analytic. Prevalence estimates of speech and MSDs in youth with 22q were compared with estimates in speakers with other complex neurodevelopmental disorders: Down syndrome, fragile X syndrome, and galactosemia.

**Results:**

Results indicated that 58.8% of the participants with 22q met criteria for speech delay, and 82.4% of the participants met criteria for MSDs, including 29.4% with speech motor delay, 29.4% with childhood dysarthria, 11.8% with childhood apraxia of speech, and 11.8% with concurrent childhood dysarthria and childhood apraxia of speech. MSDs were not significantly associated with velopharyngeal dysfunction.

**Conclusions:**

In summary, 82.4% of the participants with 22q met criteria for 1 of 4 MSDs, predominantly speech motor delay and childhood dysarthria. Cross-validation of the present findings would support viewing MSDs as a core phenotypic feature of 22q.

Also termed velocardiofacial syndrome and DiGeorge (DGR) syndrome, 22q11.2 deletion syndrome (22q) is an autosomal dominant condition associated with feeding and swallowing difficulties, speech-language delays, velopharyngeal dysfunction (VPD), cardiac defects, learning disabilities, immune deficiency, and psychiatric disorders (Bales, Zaleski, & McPherson, 2010; McDonald-McGinn et al., 1997; Rommel et al., 1999; Shprintzen et al., 1978). Relative to the present research focus, more than 90% of children with 22q present with a speech-language disorder and some degree of developmental delay (Golding-Kushner, Weller, & Shprintzen, 1985; Shprintzen et al., 1978). The syndrome occurs in one of 4,000 live births and is typically diagnosed with chromosomal microarray technologies (McDonald-McGinn & Sullivan, 2011). 22q is caused by a microdeletion on the 22nd chromosome, involving approximately 30 genes in the DGR critical region (Devriendt, Fryns, Mortier, Van Thienen, & Keymolen, 1998; Scambler et al., 1992). The distinct facial features of 22q include a long midface, malar flatness, a prominent nose and a bulbous nasal tip, narrow palpebral fissures, a thin upper lip, and decreased and/or asymmetric facial animation (Kirschner & Baylis, 2014).

Deficits of gross and fine motor skills in speakers with 22q have been well described (Van Aken et al., 2007), as have generalized motor delays, hypotonia, muscle fiber differences, and brain abnormalities (Bish, Nguyen, Ding, Ferrante, & Simon, 2004; Hultman et al., 2000; Óskarsdóttir, Persson, Eriksson, & Fasth, 2005; Zim et al., 2003). Cerebellar hypoplasia, midline anomalies (including dysgenesis of the corpus callosum), neurotransmitter abnormalities, abnormal cortical connectivity, and volumetric and morphologic abnormalities of the thalamus, basal ganglia, and temporal, frontal, and parietal lobes have also been reported (Barnea-Goraly, Eliez, Menon, Bammer, & Reiss, 2005; Barnea-Goraly et al., 2003; Bish et al., 2004; Eliez, Schmitt, White, Wellis, & Reiss, 2001; Simon, Ding, et al., 2005; Zaleski et al., 2009). Studies also suggest altered auditory processing (Rihs et al., 2013) with almost universal reports of visuospatial and spatiotemporal deficits associated with underlying structural brain and connectivity differences (Simon, 2008; Simon, Bearden, McGinn, & Zackai, 2005; Simon et al., 2008).

## Speech Deficits in Children With 22q

The extensive profile of neurodevelopmental deficits in children with 22q provides the potential explanatory context for the diverse speech findings reported in descriptive studies of children and persistent speech deficits in adults with 22q (Carneol, Marks, & Weik, 1999; D'Antonio, Scherer, Miller, Kalbfleisch, & Bartley, 2001; Kummer, Lee, Stutz, Maroney, & Brandt, 2007; Persson, Laakso, Edwardsson, Lindblom, & Hartelius, 2017; Solot et al., 2000; Zaleski et al., 2009). Findings typically include mild to severe articulation disorders and hypernasal resonance associated with VPD. Prosodic abnormalities have been described (Gorlin & Baylis, 2009), and language delay is commonly reported (Glaser et al., 2002; Niklasson, Rasmussen, Óskarsdóttir, & Gillberg, 2001; Swillen et al., 1997). These segmental, suprasegmental, and language deficits have been associated with reduced speech intelligibility that is more severe and persistent in some children than observed in some speakers with Down syndrome (DS), cleft palate, and other phenotypic overlaps (Baylis, Munson, & Moller, 2008; D'Antonio et al., 2001; Persson, Lohmander, Jönsson, Óskarsdóttir, & Söderpalm, 2003; Rakonjac et al., 2016; Scherer, D'Antonio, & Kalbfleisch, 1999).

Although research in speakers with 22q has focused on speech disorders and VPD, a few studies have reported motor speech disorders (MSDs) in speakers with 22q, including childhood dysarthria and childhood apraxia of speech (CAS; e.g., Carneol et al., 1999; D'Antonio et al., 2001; Golding-Kushner et al., 1985; Kummer et al., 2007; Mills, Gosling, & Sell, 2006; Persson et al., 2003; Rakonjac et al., 2016; Solot et al., 2000). Solot et al. (2000) anecdotally described features of flaccid dysarthria in children with 22q. Mills et al. (2006), in a retrospective study of 76 patients with 22q, reported that 36% had features of apraxia. A follow-up study of 21 of the children assessed with the Nuffield Dyspraxia Assessment (Connery, 1992) indicated that 52% had dyspraxic features; the authors noted that ascertainment bias (participation weighted by the most concerned parents) was a possible methodological concern. Kummer et al. (2007), in a retrospective chart review of 28 patients with 22q, reported that 15 (54%) of the children had a diagnosis of “apraxia” or “oral–motor dysfunction.” In the second stage of the study, 20 children with cleft palate, seven with 22q, and 47 controls were administered the Apraxia Profile (Hickman, 1997). Kummer et al. reported that children in the group with 22q had more features of apraxia and poorer intelligibility than the comparison groups of speakers with cleft palate only and cleft lip and palate. Interrater and intrarater reliability of measurements was not reported. Between-group differences indicating poorer performance of the participants with 22q were observed across multiple variables, including mean utterance length, oral movements, single-word imitation, and prosody imitation. Characteristics of dysarthria and presence and severity of VPD were not addressed.

## Research Needs in MSD in Speakers With 22q

Some methodological constraints in the speech literature on MSD in speakers with 22q include (a) reliance on retrospective, chart-level clinical data; (b) lack of validated and reliable assessment procedures for MSD; and (c) lack of a validated diagnostic criterion and method to classify a child as positive for MSD, notably for CAS (American Speech-Language-Hearing Association, 2007; McCauley & Strand, 2008). There are no studies that have examined whether children with 22q who have prior or persistent VPD have a higher prevalence of MSDs or whether other phenotypic features of 22q (e.g., hearing disorder, swallowing disorder) are associated with motor speech status. Because the etiology of VPD in speakers with 22q is multifactorial, a differential diagnosis of VPD may be more challenging and is often delayed, compared with other populations with nonsyndromic VPD (Kirschner & Baylis, 2014). Diagnosis and management of VPD can be further complicated by the co-occurrence of severe speech sound or motor speech deficits. Motor speech deficits can exacerbate the severity of perceived hypernasality, limit velopharyngeal closure, and/or negatively impact the degree of improvement after VPD surgery. Whereas studies to date have examined the origins and severity of VPD in speakers with 22q, this study examines potential associations between VPD and types of MSDs in youth with 22q.

## Statement of Purpose

A prospective study of the prevalence of MSDs in children and adolescents with 22q using contemporary methods to classify MSDs has the potential to extend the behavioral phenotype, contribute to explication of the neurogenomic substrates, and inform clinical management. The primary goal of this study was to obtain initial estimates of the prevalence of MSDs in children and adolescents with 22q using a recently extended and finalized classification system that includes five mutually exclusive motor speech classifications. The Method section includes a brief description of the classification system, termed the Speech Disorders Classification System (SDCS), which is described in detail in several technical and substantive reports (Mabie & Shriberg, 2017; Shriberg & Mabie, 2017; Shriberg, Kwiatkowski, Campbell, Mabie, & McGlothlin, 2018a; Shriberg, Kwiatkowski, Campbell, Mabie, & McGlothlin, 2018b). Prevalence findings are compared with findings using the SDCS in studies of three other complex neurodevelopmental disorders with overlapping phenotypes. Because the conceptual and procedural information on methods and measures are relatively new, there is a need to include a sufficient amount of measurement information in the present text and an appendix to understand findings in the larger context of research using the SDCS. The secondary study goal was to examine possible associations between MSD classifications and commonly studied hearing, swallowing, velopharyngeal, speech, and other developmental features of 22q.

## Method

### Participants

#### Group With 22q

Children with 22q were identified from parent responses to mailed and emailed announcements sent to patients at two large pediatric hospitals in the midwestern United States (Children's Hospital of Wisconsin [Milwaukee, WI] and Nationwide Children's Hospital [Columbus, OH]). Patient mailing lists were generated from an electronic medical record query for children with a diagnosis of 22q, velocardiofacial syndrome, or DGR syndrome, for ages 6–18 years. Study announcements were distributed to patients at each institution's 22q clinic during their routine annual clinic visit. Study announcements were also posted on 22q foundation websites and 22q social media pages. Parents then self-referred to the study and contacted the investigator if they were interested in participating and were screened for inclusion/exclusion criteria over the phone before scheduling a study visit. The recruitment protocol, assessment protocol, and consent and assent forms were approved by institutional review boards at each assessment site and the University of Wisconsin-Madison, which served as the primary data analysis site for the study. Inclusionary criteria were as follows: (a) diagnosis of 22q by fluorescence in situ hybridization testing or microarray, (b) age of 6–18 years, (c) English as the participant's primary language, and (d) no history of permanent bilateral hearing loss. Twenty children and adolescents met these study criteria and were assessed on the protocol to be described. Because three of the 20 children did not produce a conversational speech sample sufficient for motor speech classification, findings to be reported are based on a sample of 17 children and adolescents aged 5–18 years with 22q.

#### Comparison Groups

Conversational speech samples were available from three comparison groups totaling 104 participants studied in associated research in speech sound disorders. The three groups included 45 participants, 8–20 years old, with DS (Wilson, Abbeduto, Camarata, & Shriberg, 2018); 28 participants, 10–21 years old, with fragile X syndrome (FXS; Abbeduto et al., 2003); and 31 participants, 4–16 years old, with galactosemia (GAL; Shriberg, Potter, & Strand, 2011). As described later in Table 1, participants in these three groups were in the same approximate age range as the present participants with 22q, had a high prevalence of MSDs, and had medical issues and developmental delays, and participants with DS were at a similar greater risk for conductive hearing loss. Recruitment procedures were generally similar to those described for participants in the group with 22q, including announcements to parent groups and referrals to the study from local speech-language pathologists. Assessment methods for participants in the three comparison groups were generally similar to those to be described for the children with 22q, and the perceptual and acoustic data reduction methods to be described were the same and completed by the same research personnel.

**Table 1.** Demographic, intelligence, language, and speech characteristics of 17 participants with 22q11.2 deletion syndrome (22q) and 104 participants in three comparison samples of children and youth with complex neurodevelopmental disorders (DS = Down syndrome; FXS = fragile X syndrome; GAL = galactosemia).

Variable	Complex neurodevelopmental disorders						Data and standard scores			Percentile scores			*z* Scores^a^		
	22q	DS	FXS	GAL	*n*	%	*M*	*SD*	Range	*M*	*SD*	Range	*M*	*SD*	Range
Demographic															
Chronological age (years)	X				17		10.3	3.3	5–18						
		X			45		14.1	2.2	10–20						
			X		28		16.0	3.2	11–22						
				X	31		8.8	2.9	5–16						
Male	X				11	64.7									
		X			25	55.6									
			X		28	100.0									
				X	20	64.5									
Intelligence															
IQ composite^b^	X				17		77.8	11.5	54–100	11.8	12.5	1–50			
		X			46		44.0	8.3	36–79	^—^	^—^	^—^			
			X		27		38.3	5.4	36–57	^—^	^—^	^—^			
				X	30		86.8	16.9	40–111	^—^	^—^	^—^			
Language															
Oral composite^c^	X				16		77.4	11.8	62–103	11.9	15.7	1–58			
		X			14		42.6	4.4	40–56	0.1	0.0	0.1–0.2			
			X		^—^		^—^	^—^	^—^	^—^	^—^	^—^			
				X	31		79.6	15.3	40–114	^—^	^—^	^—^			
Speech															
Sounds-in-Words^d^	X				17		73.6	27.4	40–106	11.6	13.2	1–42			
		X			^—^		^—^	^—^	^—^	^—^	^—^	^—^			
			X		^—^		^—^	^—^	^—^	^—^	^—^	^—^			
				X	^—^		^—^	^—^	^—^	^—^	^—^	^—^			
Percentage of consonants correct	X				17		82.4	13.3	61.1–97.2				−4.27	1.52	−5.00 to −0.19
		X			45		78.9	8.7	59.3–93.3				−4.96	0.26	−5.00 to −3.29
			X		28		93.0	3.3	84.8–98.8				−4.6	1.1	−5.00 to −0.93
				X	31		84.3	13.2	46.5–98.5				−3.44	1.81	−5.00 to 0.33
Percentage of vowels correct	X				17		92.6	7.4	76.0–99.2				−4.27	1.55	−5.00 to −0.01
		X			45		89.9	4.3	77.7–96.7				−5.00	0.00	*
			X		28		96.2	2.3	88.4–99.0				−4.87	0.49	−5.00 to −2.90
				X	31		92.4	7.8	69.9–99.7				−3.76	1.84	−5.00 to 0.35
Intelligibility Index (%)	X				17		93.9	8.6	65.9–99.5				−3.22	1.82	−5.00 to −0.14
		X			45		81.3	12.3	50.1–99.0				−4.94	0.43	−5.00 to −2.12
			X		28		84.4	12.4	50.4–98.0				−4.91	0.34	−5.00 to −3.26
				X	31		93.2	11.2	54.8–100.0				−3.01	2.13	−5.00 to 0.90

*Note. z* Score values for the speech variables were truncated at −5.00 *SD* units. Blank cells indicate not applicable; cells with em dashes indicate data not available.

a Using the age and sex reference data in “Reference Data for the Madison Speech Assessment Protocol (MSAP): A Database of 150 Participants 3-to-18 Years of Age with Typical Speech (Tech. Rep. No. 18)” N. L. Potter, S. Hall, H. B. Karlsson, M. Fourakis, H. L. Lohmeier, J. L. McSweeny, … L. D. Shriberg, 2012, Phonology Project, Waisman Center, University of Wisconsin-Madison.

b Standard scores: Kaufman Brief Intelligence Test–Second Edition (Kaufman & Kaufman, 2004); Stanford–Binet Intelligence Scale–Fourth Edition (Thorndike et al., 1986); Wechsler Intelligence Scale for Children–Third Edition (Wechsler, 1991); and Wechsler Abbreviated Scale of Intelligence–Second Edition (Wechsler, 2011).

c Standard scores: Oral and Written Language Scales (Carrow-Woolfolk, 1995).

d Standard scores: Goldman-Fristoe Test of Articulation*–*Second Edition (Goldman & Fristoe, 2000).

* Not appropriate.

### Assessment

Testing of participants with 22q was completed in quiet examination rooms at each of the two sites by the first author a certified and licensed speech-language pathologist with experience in pediatric craniofacial speech disorders, including 22q. Parents or guardians of participants provided informed consent for their child to participate in the study. Children aged 9 years and older were also asked to sign an assent form. Assessment sessions lasted approximately 2 hr, typically completed in one session. Participants were paid a small stipend for completing the study.

The Madison Speech Assessment Protocol (Shriberg et al., 2010a) was administered to all participants. The protocol includes the Kaufman Brief Intelligence Test–Second Edition (Kaufman & Kaufman, 2004), the Oral and Written Language Scales (OWLS; Carrow-Woolfolk, 1995), a parental questionnaire describing a participant's medical and speech treatment history, the Goldman-Fristoe Test of Articulation–Second Edition (GFTA-2; Goldman & Fristoe, 2000), and 15 speech tasks ranging from single-word imitation tasks to a conversational speech sample. An oral–motor examination was also completed by the site investigator (the first author) to document facial, lip, and tongue mobility and symmetry and intraoral observations. For the conversational sample, the examiner asked participants to answer and elaborate on questions about themselves and activities of daily life, with all questions introduced in a similar manner appropriate for participants' estimated cognitive level (McSweeny, 1998; Shriberg & Kwiatkowski, 1985). Responses to the Madison Speech Assessment Protocol speaking tasks were digitally recorded on a Marantz Professional recorder (CDR420) with an external desktop microphone (Shure Microflex MX412D/C) positioned approximately 8 in. from the participant's mouth or a comparable system. Assessments of participants in the three comparison groups, as described in the reference citations, were completed using a similar protocol and comparable recording methods. Samples originally recorded on high-quality analog recorders were digitized for analyses using Adobe Audition 2.0 and conventional procedures. Last, parents completed an extensive questionnaire on the child's medical, developmental, and therapy history as well as provided input regarding their perceptions of their child's speech.

### Speech and Motor Speech Measures and Classification Criteria

Table 2 includes the five speech and motor speech classifications in the finalized version of the SDCS (Shriberg et al., 2018a) and brief descriptions of the measures used to classify speakers as positive for each classification. The operationalized perceptual and acoustic signs of each speech and motor speech classification in the SDCS are standardized using databases of 150 typical speakers, 3–18 years of age (Potter et al., 2012), and 50 typical speakers, 20–80 years of age (Scheer-Cohen et al., 2013).

**Table 2.** Speech classifications, motor speech classifications, and dysarthria subtype classifications in the Speech Disorders Classification System (SDCS).

SDCS		Age (years;months) at assessment	Description	Reference^a^
Classes, types, and subtypes	Abbreviation			
Five speech classification types				
Normal(ized) speech acquisition	NSA	3–80	Does not meet criteria for any of the four speech disorder classifications; includes children 3–8 years old with only distortion errors	2, 3, 4
Speech errors	SE	6–8;11	Age-inappropriate speech sound distortions	3, 4
Persistent speech errors	PSE	9–80	Age-inappropriate speech sound distortions that persist past 9 years of age	4, 5
Speech delay	SD	3–8;11	Age-inappropriate speech sound deletions and/or substitutions	3, 4
Persistent speech delay	PSD	9–80	Age-inappropriate speech sound deletions and/or substitutions that persist past 9 years of age	3, 4, 5
Five motor speech classification types				
No motor speech disorder	No MSD	3–80	Does not meet criteria for any of the four motor speech disorder classifications	2, 6, 8
Speech motor delay	SMD	3–80	Meets Precision–Stability Index criterion for SMD	2, 6, 8
Childhood dysarthria	CD	3–80	Meets Dysarthria Index and Dysarthria Subtype Indices criteria for CD	2, 6, 8
Childhood apraxia of speech	CAS	3–80	Meets Pause Marker criteria for CAS	6, 7, 8, 9
Childhood dysarthria and childhood apraxia of speech	CD & CAS	3–80	Meets SDCS criteria for CD & CAS	2, 6, 8
Five dysarthria subtypes				
Ataxic		3–80	Cerebellar disorder	1, 2
Spastic		3–80	Upper motor neuron disorder	1, 2
Hyperkinetic		3–80	Basal ganglia disorder; increased movement	1, 2
Hypokinetic		3–80	Basal ganglia disorder; decreased movement	1, 2
Flaccid		3–80	Lower motor neuron disorder	1, 2

*Note.* The five speech classifications are mutually exclusive, as are the five motor speech classifications. The five subtypes of dysarthria classifications are not mutually exclusive. That is, a speaker can meet percentile criteria for more than one of the five listed dysarthria subtype classifications (i.e., mixed dysarthria). See Appendix B for the measures used to classify each motor speech disorder.

a 1: Duffy (2013); 2: Mabie and Shriberg (2017); 3: Shriberg (1993); 4: Shriberg et al. (1997); 5: Shriberg et al. (2010a); 6: Shriberg and Mabie (2017); 7: Shriberg et al. (2017a); 8: Shriberg et al. (2018a); and 9: Tilkens et al. (2017).

The first section in Table 2 provides information about each of the five SDCS speech classifications, including the title, abbreviation, eligible age range at assessment, description, and references. The second section in Table 2 includes similar information for each of the five SDCS motor speech classifications, including reference to technical reports that provide reference data for the diagnostic markers of MSDs used in this study (Mabie & Shriberg, 2017; Shriberg & Mabie, 2017). In addition to dysarthria and apraxia, the SDCS includes a third MSD termed *speech motor delay*. Speech motor delay (Shriberg et al., 2018a) has recently been proposed as a delay in speech motor development, similar to the psychometric construct of delays in other verbal traits (i.e., speech delay, language delay, reading delay). Speech motor delay provides a classification entity for children with spatiotemporal delays in the precision and stability of speech, prosody, and voice that do not meet criteria for childhood dysarthria or CAS.

The third section of Table 2 includes information on the five SDCS subtypes of dysarthria. The dysarthria subtypes are based on Duffy's (2013) descriptions of research and clinical applications of the Mayo Clinic classification system for adult neurogenic speech disorders and from research in pediatric speech sound disorders (Mabie & Shriberg, 2017). Appendix A provides a list of frequently used abbreviations. Appendix B includes descriptions of each of the three measures used to make the motor speech classifications, including a copy of the Precision–Stability Index (Mabie & Shriberg, 2017) used to identify speech motor delay and a copy of the Dysarthria Index/Dysarthria Subtypes Indices (Mabie & Shriberg, 2017) used to identify childhood dysarthria and subtypes of dysarthria and comorbid childhood dysarthria and CAS. Extended descriptions and procedures for each of the 32 items in the Precision-Stability Index and the 34 items in the Dysarthria Index/Dysarthria Subtypes are provided in tables for each measure in Appendix B. Appendix B also includes a description of and references for the Pause Marker, the perceptual–acoustic marker to identify CAS.

### Data Reduction and Reliability Estimates

#### Transcription, Prosody-Voice Coding, and Acoustic Analyses

Four research transcriptionists and acoustic analysts completed narrow phonetic transcription, prosody-voice coding, and acoustic analyses of the continuous speech samples and speech tasks to be described in the Results section. A research assistant error-checked the transcripts and entered them into a software suite titled Programs to Examine Phonetic and Phonologic Evaluation Records (PEPPER, 2019).

Table 3 includes estimates of the reliability of the speech, prosody-voice, and acoustic variables to be reported. Findings are based on a randomly selected 24.0% of the conversational speech samples from participants with 22q, including averaged interjudge data. The interjudge and intrajudge percentages of agreement values in Table 3 are consistent with those reported in prior studies using similar perceptual and acoustic methods, ranging from a few estimates in the 60% range to most estimates in the 70%–90% range (Shriberg et al., 2010b).

**Table 3.** Reliability estimates for interjudge and intrajudge agreement for phonetic transcription, prosody-voice coding, and acoustic analyses.

Data	Agreement types		No. of tokens analyzed	Variable	Percentage of agreement
	Interjudge	Intrajudge			
Phonetic transcription	X			Consonants	
			322 utterances; 789 words	Broad	89.1
				Narrow	68.2
				Vowels	
				Broad	82.2
				Narrow	71.4
		X		Consonants	
			322 utterances; 794 words	Broad	93.7
				Narrow	81.8
				Vowels	
				Broad	88.0
				Narrow	79.4
Prosody-voice coding	X		95 utterances	Appropriate–inappropriate	88.0
		X		Appropriate–inappropriate	92.6
Acoustic analyses	X			Phoneme duration	
			79 Consonants	Consonants	85.6
			309 Vowels	Vowels	84.9
		X		Phoneme duration	
			90 Consonants	Consonants	83.8
			305 Vowels	Vowels	87.5
	X			Vowel frequency	
			312 Vowels	F0	97.7
			51 Vowels	F1	92.8
			43 Vowels	F2	79.8
		X		Vowel frequency	
			306 Vowels	F0	97.9
			54 Vowels	F1	91.8
			49 Vowels	F2	95.7
	X			Pause variables	
			52 Pauses	Pause–nonpause	92.3
			48 Pauses	Appropriate–not appropriate	60.4
			11 Pauses	Type 1–Type 2	90.9
		X		Pause variability	
			52 Pauses	Pause–nonpause	90.4
			47 Pauses	Appropriate–not appropriate	66.0
			10 Pauses	Type 1–Type 2	80.0

#### VPD Ratings

The first author completed perceptual ratings of variables associated with VPD for each of the participants with 22q. Audio-recorded conversational speech samples were rated using the Cleft Audit Protocol for Speech-Augmented–Americleft Modification rating scales for hypernasality, hyponasality, and audible nasal emission (ANE)/nasal turbulence (see Chapman et al., 2016, for a review). Four (23.5%) of the participants in the group with 22q were randomly selected for an estimate of intrarater reliability of the perceptual ratings. For an estimate of the interrater reliability, all 17 conversational speech samples from the participants with 22q were rated by a colleague with speech-language pathology qualifications in craniofacial populations similar to those of the first author. Intrarater reliability estimated with intraclass correlation coefficients was .91 for hypernasality, .80 for ANE, and .67 for hyponasality. Interrater reliability was .89 for hypernasality, .88 for ANE, and .65 for hyponasality. These reliability estimates are similar to those reported in other studies of perceptual speech ratings in children with craniofacial conditions (e.g., Chapman et al., 2016).

### Demographic, Cognitive-Language, and Speech Status of Participants With 22q and Comparison Participants

Table 1 includes descriptive information for participants in the group with 22q and the three comparison groups. Chronological age across the four groups ranged from 5 to 22 years, averaging 8.8–16.0 years. As reported for many neurodevelopmental disorders, the proportion of males was higher than the proportion of females in each of the four groups, ranging from 55% to 100%. The wide ranges of IQ findings across the four groups (36–111) and percentile scores for participants in the group with 22q (1st–50th percentiles) are consistent with the heterogeneity described in the literatures on each disorder. Language findings in Table 1 also indicated a wide range of performance (standard scores of 40–114) across the four groups, with percentile findings for participants in the group with 22q similar to their IQ percentile findings. For the prevalence questions posed in the present article, standardized classification of speech and motor speech status is referenced to participants' chronological age and sex, rather than to nonverbal or language age.

Findings for the four speech measures in Table 1 are consistent with literature reports of significant prevalence of speech disorders in each group. For participants in the group with 22q, average percentile scores on the Sounds-in-Words section of the GFTA-2 (Goldman & Fristoe, 2000) are similar to their average percentile scores on the IQ and language measures. For this target group and each of the comparison groups, the range and magnitudes of *z* score findings for the percentage of consonants correct, percentage of vowels correct, and Intelligibility Index (Shriberg, 1993; averaging approximately 3–5 *SD* units lower than typical speakers of the same age and sex) document the significant speech deficits reported in participants within each of the four groups.

## Results

### Prevalence of Speech Disorders and MSDs in Participants With 22q

**Figure 1.**
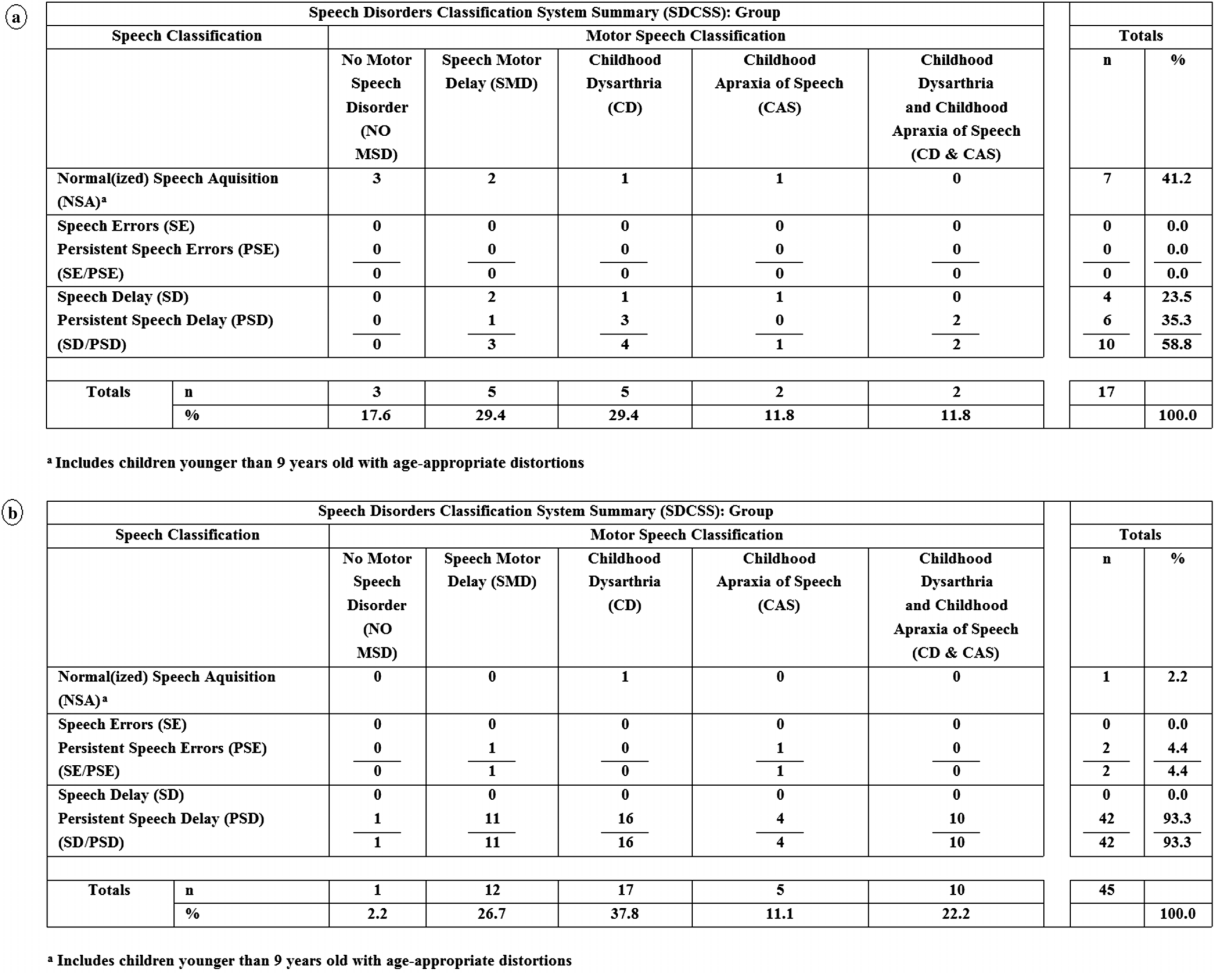
Estimates of the prevalence of speech and motor speech disorders in 17 persons with 22q11.2 deletion (a) and 45 persons with Down syndrome (b).

**Figure 2.**
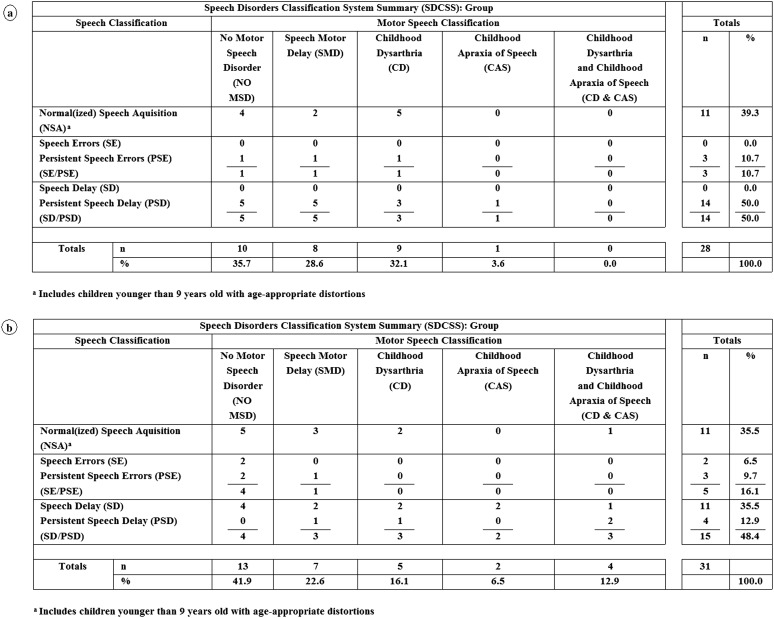
Estimates of the prevalence of speech and motor speech disorders in 28 persons with fragile X syndrome (a) and 31 persons with galactosemia (b).

**Figure 3.**
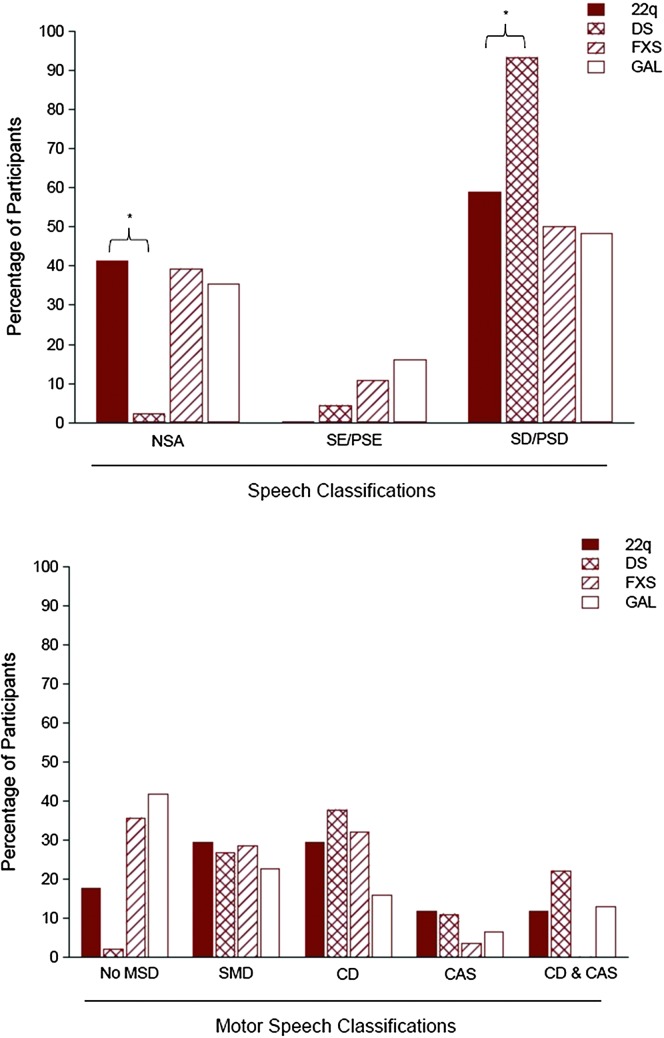
Descriptive and inferential statistics comparing 22q11.2 deletion syndrome (22q) speech and motor speech prevalence findings with findings for participants with one of the other three complex neurodevelopmental disorders in Figures 1 and 2. DS = Down syndrome; FXS = fragile X syndrome; GAL = galactosemia; NSA = normal(ized) speech acquisition; SE = speech errors; PSE = persistent speech errors; SD = speech delay; PSD = persistent speech delay; MSD = motor speech disorder; SMD = speech motor delay; CD = childhood dysarthria; CAS = childhood apraxia of speech.

Figures 1 and 2 include findings from an analytic termed the *SDCS Summary* (SDCSS). The SDCSS cross-classifies a speaker or a group of speakers' speech status and motor speech status using the mutually exclusive classifications described in Table 2. The SDCSS in Figure 1a includes the speech and motor speech classifications' prevalence findings for participants with 22q. Figures 1b, 2a, and 2b, respectively, include SDCSS prevalence findings for participants with DS, FXS, and GAL. Figure 3 includes both descriptive statistical findings for the prevalence percentages in Figures 1 and 2 and the inferential statistical findings comparing 22q speech and motor speech prevalence findings with findings for each of the three other comparison groups. The following sections describe these findings.

### Speech Classification Findings

The first set of findings in the four panels in Figures 1 and 2 are age–sex standardized (Shriberg, Austin, Lewis, McSweeny, & Wilson, 1997) prevalence estimates for the five speech classifications for each of the four participant groups. As shown in Figure 3, there were two statistically significant differences between the percentages of participants in the group with 22q compared with percentages for that classification in each of the other three groups. Whereas 41.2% of the participants in the group with 22q had normal or normalized speech at assessment, only 2.2% of the participants in the group with DS had normal(ized) speech at assessment (Fisher's exact test of proportions = 0.000). Moreover, whereas 58.8% of the participants in the group with 22q met criteria for speech delay or persistent speech delay at assessment, 93.3% of the participants in the group with DS met criteria for speech delay or persistent speech delay (Fisher's exact test of proportions = 0.003). Thus, the clinically high prevalence of speech delay or persistent speech delay in the present sample of adolescents with 22q was significantly lower than the prevalence of speech delay or persistent speech delay in the comparison sample of adolescents with DS but did not differ significantly from the prevalence of speech delay or persistent speech delay in the sample with FXS or GAL.

### Motor Speech Classification Findings

Figures 1–3 also include findings for the primary question posed in this report: Estimates of the prevalence of each of the five motor speech classifications are defined in Table 2. As shown in Figure 1a, 17.6% of the participants in the group with 22q had no MSD at assessment, as determined from age–sex standardized perceptual and acoustic reference data (Potter et al., 2012; Scheer-Cohen et al., 2013) for the measures described in Appendix B. The remaining 82.4% of participants met criteria for one of four classifications of MSDs, including speech motor delay (29.4%), childhood dysarthria (29.4%), CAS (11.8%), and childhood dysarthria and CAS (11.8%). As indicated in Figure 3, none of these prevalence estimates for the motor speech classifications differed significantly from those of participants in the samples with DS, FXS, or GAL.

### Cross-Classification Findings

As shown in the cross-classification findings for participants with 22q (see Figure 1a), speech classifications and motor speech classifications can be independent of one another. Among the seven participants with 22q who had normal or normalized speech acquisition (NSA) at assessment (which can include age-inappropriate speech sound distortions, but not age-inappropriate speech sound deletions or substitutions), three participants met criteria for no MSD, and the remaining four met criteria for one of the motor speech classifications. Each of the 10 participants who met criteria for speech delay or persistent speech delay met criteria for one of the four motor speech classifications. A substantial percentage of children with 22q had early or persistent speech delay, and a larger percentage had early or persistent MSD (see Figure 1a). With the exception of the two statistically significant findings in Figure 3, the estimated prevalence of speech disorders and MSDs in participants with 22q was comparable with estimates in samples of children and adolescents with the three other types of complex neurodevelopmental disorders.

### Prevalence Estimates for Dysarthria Subtypes in Participants With 22q

As indicated in Figures 1a and 3, childhood dysarthria (i.e., the total number of participants with childhood dysarthria and those with both childhood dysarthria and CAS) was the most prevalent type of MSD within the group with 22q. Seven (41.2%) of the participants in the group with 22q met the classification criteria for childhood dysarthria, either alone (five participants) or concurrent with CAS (two participants). Findings were generally similar in each of the three comparison groups (see Figure 3).

Analyses of findings from the Dysarthria Subtype Indices shown in Appendix B were completed to determine if any one or more subtypes of dysarthria were characteristic of all or most of the seven participants with childhood dysarthria with or without concurrent CAS. Findings were inconclusive. At least one of the seven participants had percentile scores at or below the 10th percentile on three of the dysarthria subtypes listed in Appendix B, including hyperkinetic, hypokinetic, and flaccid. Thus, there were too few data for a meaningful interpretation of subtype findings.

### Motor Speech Classifications in Participants With 22q in Relation to Developmental and Other Variables

Tables 4 [Table T5] [Table T6]–7 include information on cognitive, language, medical, developmental, VPD, and speech variables organized by participants' motor speech classifications. Findings were obtained from parent reports, medical records' review, and direct observations at assessment. Cell sizes are too small for inferential statistical analyses. Overall, there did not appear to be a strong association between motor speech classifications and developmental or other variables in the three tables, but findings may generate hypothetical–deductive questions to pose in the future.

**Table 4.** Motor speech classifications in group participants with 22q11.2 deletion syndrome and status on hearing, swallowing, motor, and cognition variables.

Participants			Hearing		Swallowing	Motor		Cognition	
No.	Motor speech classification	Speech classification	Ventilation tubes	Hearing loss	History of swallowing problems	History of gross or fine motor difficulties	Motor observations: lips^a^	Cognitive disability	Learning disability
7	No MSD	NSA	+	+	+	+		−	−
8	No MSD	NSA	−	−	+	−	1, 2	+	+
17	No MSD	NSA	+	+	+	+		+	+
2	SMD	PSD	NR	−	−	+	2	+	+
5	SMD	SD	+	−	+	+		−	−
10	SMD	NSA	+	−	+	+	2	−	+
12	SMD	NSA	+	−	−	−	2	−	−
15	SMD	SD	+	+	+	+		−	−
1	CD	PSD	NR	−	NR	+		−	−
6	CD	SD	NR	−	−	+	1	−	−
11	CD	PSD	NR	−	+	+		+	+
14	CD	NSA	+	+	−	+		+	+
16	CD	PSD	−	−	−	−		−	−
3	CAS	SD	−	−	−	+	2	−	−
9	CAS	NSA	+	−	+	+		+	+
4	CD&CAS	PSD	+	+	–	+	2	+	+
13	CD&CAS	PSD	+	−	NR	+	2	+	−

*Note.* No MSD = no motor speech disorder; NSA = normal(ized) speech acquisition; + = yes; − = no; SMD = speech motor delay; PSD = persistent speech delay; NR = not reported/unknown; SD = speech delay; CD = childhood dysarthria; CAS = childhood apraxia of speech.

a 1 = reduced movement; 2 = asymmetrical movement.

**Table 5. T5:** Motor speech classifications in group participants with 22q11.2 deletion syndrome and individual demographic, IQ, language, and speech data.

No.	Motor speech classification	Speech classification	Age (years)	Sex	KBIT-2 IQ composite SS	OWLSLC SS	OWLSOE SS	OWLSOC SS	GFTA-2 SS
7	No MSD	NSA	7	M	84	94	77	84	97
8	No MSD	NSA	13	M	54	63	73	66	104
17	No MSD	NSA	7	F	86	89	93	90	82
2	SMD	PSD	9	M	70	72	57	62	52
5	SMD	SD	9	M	75	72	76	72	< 40
10	SMD	NSA	15	F	86	96	110	103	103
12	SMD	NSA	9	F	84	62	85	72	99
15	SMD	SD	5	M	80	84	74	77	51
1	CD	PSD	12	M	73	71	66	66	< 40
6	CD	SD	7	M	85	97	83	89	45
11	CD	PSD	18	F	63	75	68	70	82
14	CD	NSA	12	F	77	62	67	62	101
16	CD	PSD	9	F	100	94	94	93	106
3	CAS	SD	8	M	91	81	82	80	68
9	CAS	NSA	12	M	68	80	71	79	101
4	CD&CAS	PSD	11	M	83	81	71	74	< 40
13	CD&CAS	PSD	10	M	64	DNT	DNT	DNT	< 40

*Note.* Participant 13 did not complete the OWLS due to time constraints. KBIT-2 IQ Composite SS = Kaufman Brief Intelligence Test–Second Edition, IQ composite standard score; OWLS = Oral and Written Language Scales (SS = standard score; LC = listening comprehension; OE = oral expression; OC = oral composite); GFTA-2 SS = Goldman-Fristoe Test of Articulation–Second Edition, standard score; No MSD = no motor speech disorder; NSA = normal(ized) speech acquisition; SMD = speech motor delay; PSD = persistent speech delay; SD = speech delay; CD = childhood dysarthria; CAS = childhood apraxia of speech; DNT = did not test; M = male; F = female.

**Table 6. T6:** Motor speech and speech classifications in group participants with 22q11.2 deletion syndrome and individual data on speech history, velopharyngeal dysfunction, and speech ratings at assessment.

Participants			Speech history		Velopharyngeal dysfunction			Speech ratings^a^		
No.	Motor speech classification	Speech classification	Speech therapy^b^	Age at onset of therapy	Management	Current or past signs^b^	Current severity^c^	Hypernasality	Hyponasality	ANE
7	No MSD	NSA	+	3 mos	Pharyngeal flap	+	2	2	1	0
8	No MSD	NSA	+	4 yrs	None	−	1	0	0	0
17	No MSD	NSA	NR	NR	Pharyngeal flap	+	2	2	0	2
2	SMD	PSD	+	3.5 yrs	Pharyngeal flap	+	2	2	0	2
5	SMD	SD	+	2 yrs	None	+	2	3	0	1
10	SMD	NSA	+	2 yrs	Palate repair	+	2	2	0	0
12	SMD	NSA	+	NR	None	+	1	1	1	0
15	SMD	SD	+	9 mos	None	−	1	0	0	0
1	CD	PSD	+	2.5 yrs	Palatal lift	+	1	0	0	1
6	CD	SD	+	1 yr	Pharyngeal flap	+	2	3	0	2
11	CD	PSD	+	6 yrs	None	+	1	1	0	1
14	CD	NSA	+	birth	Pharyngeal flap	+	1	0	0	1
16	CD	PSD	+	4 yrs	Pharyngeal flap	+	2	2	0	2
3	CAS	SD	+	7 mos	Sphincter pharyngoplasty	+	1	1	0	1
9	CAS	NSA	+	9 mos	Pharyngeal flap	+	1	1	1	0
4	CD&CAS	PSD	+	20 mos	Pharyngeal flap	+	3	4	1	1
13	CD&CAS	PSD	NR	NR	None	−	1	0	2	0

*Note.* No MSD = no motor speech disorder; NSA = normal(ized) speech acquisition; mos = months; yrs = years; NR = not reported/unknown; SMD = speech motor delay; PSD = persistent speech delay; SD = speech delay; CD = childhood dysarthria; CAS = childhood apraxia of speech; ANE = audible nasal emission.

a Speech ratings were based on conversational speech samples using the Cleft Audit Protocol for Speech-Augmented–Americleft Modification Rating Scales. Hypernasality: 0 = absent, 1 = borderline/minimal with some perceptible increase in nasal resonance, 2 = mild with hypernasality evident on high vowels, 3 = moderate with hypernasality evident on vowels, and 4 = severe with increased nasal resonance on vowels and voiced consonants. Hyponasality: 0 = absent, 1 = mild with partial denasalization of nasal consonants, and 2 = marked with denasalization of nasal consonants and adjacent vowels. ANE/nasal turbulence (accompanying target consonant): 0 = absent, 1 = occasionally/seldom noted, and 2 = frequently noted.

b + = yes; − = no.

c 1 = minimal to none: hypernasality rating of 0 or 1 and ANE rating of 0 or 1; 2 = mild to moderate: hypernasality rating of 2 or 3, with or without ANE rating of 1 or 2; 3 = severe: hypernasality rating of 4 with ANE rating of 1 or 2.

**Table 7.** Motor speech classifications in group participants with 22q11.2 deletion syndrome and parent perceptions of their child's speech clarity, rate, willingness to talk, and willingness to provide a repetition.

No.	Motor speech classification	Speech classification	How often speech is understood by parent^a^	Child doesn't talk as well as peers	Talks too fast	Talks too slow	Child's willingness to talk^b^	Child's willingness to repeat if not understood^c^
7	No MSD	NSA	1	Y	N	N	0	2
17	No MSD	NSA	1	Y	Y	N	0	1
2	SMD	PSD	1	Y	N	N	0	2
5	SMD	SD	1	Y	N	N	2	0
12	SMD	NSA	2	Y	N	N	0	0
15	SMD	SD	1	Y	N	N	0	0
1	CD	PSD	1	Y	Y	N	0	0
6	CD	SD	1	Y	N	N	0	1
11	CD	PSD	0	Y	N	N	0	0
14	CD	NSA	0	Y	N	N	1	1
16	CD	PSD	1	Y	N	N	0	0
3	CAS	SD	1	Y	Y	N	1	1
9	CAS	NSA	1	Y	Y	N	1	2
4	CD&CAS	PSD	1	Y	N	N	0	1
13	CD&CAS	PSD	1	Y	Y	N	0	0

*Note.* No MSD = no motor speech disorder; NSA = normal(ized) speech acquisition; Y = yes; N = no; SMD = speech motor delay; PSD = persistent speech delay; SD = speech delay; CD = childhood dysarthria; CAS = childhood apraxia of speech.

a How often speech understood by parent: 3 = *never*, 2 = *sometimes*, 1 = *usually*, and 0 = *always*.

b Parent perception of child's willingness to talk: 0 = *usually willing*, 1 = *hesitant in many situations*, and 2 = *hesitant in most situations*.

c Parent perception of child's willingness to repeat if not understood: 0 = *usually willing*, 1 = *often unwilling*, and 2 = *always unwilling*. Parents of Participants 8 and 10 did not provide a response to this section of the parent questionnaire.

#### Hearing

As shown in Table 4, 10 of the 13 participants for whom information on pressure equalization tubes was available had histories of tube insertions, which is consistent with the literature on 22q. Of those participants, five (50%) also had at least one report of fluctuant conductive hearing loss. The importance of this finding for the present concern is that fluctuant conductive hearing loss due to early recurrent otitis media with effusion has been associated with types of uncommon speech errors in children with idiopathic speech delay, including initial consonant deletions, backing of stops and fricatives, and errors on glides (see Tables 1–4 in Shriberg, 2010;). Although none of these variables is diagnostic of MSDs, such uncommon and sometimes persistent errors can be mistakenly interpreted as support for motor speech deficits in young children. Research with participants with high comorbidity of both hearing loss and MSDs, such as participants with 22q, can provide opportunities to study causal substrates and phenotypic differences associated with heterogeneities in speech sound disorders.

#### Swallowing

The swallowing histories of participants with 22q in Table 4 do not support an association of the sensorimotor deficits in swallowing problems with sensorimotor deficits that define MSDs. All three of the participants with no MSD had positive histories of swallowing problems, based on parent report, and for the remaining participants with MSDs, negative histories of swallowing problems were more prevalent than positive histories.

#### Motor

Both the historical information on gross and fine motor difficulties and the observational data on lip movements in participants with 22q at assessment in Table 4 could be interpreted as consistent with the substantial prevalence of MSDs in these speakers. Fourteen of the 17 (82.4%) participants had positive histories for gross or fine motor difficulties, and eight (47.1%) were observed to have reduced and/or asymmetrical lip movements at assessment. Discussion of domain-general versus domain-specific perspectives on speech motor movements in comparison with deficits in gross motor, fine motor, and oral–motor movements (e.g., Bunton, 2008; Kent, 2015; Maas, 2017; Moore & Ruark, 1996; Ziegler & Ackermann, 2013) is beyond the scope of this study.

#### IQ, Language, and Articulation

Data provided in Tables 4 and 5 include findings for cognitive-language variables within each motor speech classification. Only four children with 22q (23.5%) had no evidence of cognitive or learning disabilities, and these participants did not cluster in any particular motor speech category. Many children had IQ and/or language performance in the borderline to mildly impaired range, consistent with the 22q literature reporting a mean IQ in the mid-70s (Woodin et al., 2001). There was no clear relationship between cognitive–linguistic and articulation variables. Age at testing was negatively correlated with OWLS oral composite scores (*r* = −.49), and Kaufman Brief Intelligence Test–Second Edition IQ composite scores were significantly correlated with all OWLS scores (listening comprehension: *r* = .60, oral expression: *r* = .88, oral composite: *r* = .61).

#### Speech History

Consistent with the standardized speech and intelligibility scores in Table 1, all (100%) of the 15 participants with available parent information on speech history in Table 6 reported histories of speech-language therapy, beginning with swallowing/feeding therapy and other types of early intervention in the first year of life and continuing in the present assessment data for as long as 6 years. There was no apparent association between months/years of speech therapy and the five motor speech classifications at assessment.

#### VPD

Eleven of the 17 (64.7%) participants with 22q in Table 6 had histories of procedures to normalize or mitigate VPD. These included participants in each of the five motor speech classifications. Neither the type of management nor the past or current signs of VPD were associated with the type of MSD. Notably, three participants with NSA and no MSD had ANE ratings of 1 or 2 (see Table 6, footnote b) at assessment.

#### Speech Ratings

As shown in Table 6, neither hypernasality, hyponasality, nor ANE ratings at assessment were associated with speech motor classifications. Consistent with VPD findings in the earlier 22q literature review, hypernasality was the most prevalent deficit in participants in each of the motor speech classifications. As with the findings for gross and fine motor problems (see Table 4), the sensorimotor substrates of hypernasality and nasal emission were not significantly associated with the putative neuromotor substrates of speech motor delay, childhood dysarthria, CAS, or comorbidity of the latter two disorders, as assessed with the present behavioral measures.

#### Parent-Reported Speech Characteristics

As shown in Table 7, parents of 15 of the 17 children in the group with 22q provided input regarding their perceptions of their child's speech and their child's response to intelligibility challenges. Furthermore, 93.3% of parents reported that they always or usually understood their child's speech, but 100% also reported that their child did not talk as well as peers. None (0%) of the parents believed that their child talked too slowly; 33.3% believed that their child talked too fast. Last, although 73.3% of the children reportedly were always willing to talk, 53.3% of the children were always or often unwilling to repeat themselves if not understood.

## Discussion

Findings indicated that 58.8% of this sample of persons with 22q met perceptual criteria for early or persistent speech disorder, as defined by age-inappropriate deletion and substitution errors. Findings also indicated that 82.4% of the participants met standardized perceptual and acoustic criteria for one of four classifications of MSDs. On the later finding, approximately 30% (29.4) of the participants met criteria for speech motor delay, approximately 30% (29.4) met criteria for childhood dysarthria, approximately 12% (11.8) met criteria for CAS, and approximately 12% (11.8) met criteria for childhood dysarthria and CAS. At the power levels available for the present sample sizes, there were no statistically significant differences in the prevalences of each of the four types of MSDs in participants with 22q compared with prevalence estimates in participants with DS, FXS, and GAL assessed with the same measures. There also were no strong associations between the motor speech classifications of participants with 22q and variables indexing their demographic, cognitive–linguistic, developmental, and VPD status. The following discussions address research and clinical considerations of findings for each type of MSD in youth with 22q.

### Speech Motor Delay in Speakers With 22q

As cited previously, speech motor delay has only recently been proposed as a classification entity for children with spatiotemporal delays in the precision and stability of speech, prosody, and voice. Speech-timing deficits affecting the velopharyngeal mechanism have been reported in some children with 22q (Baylis, Watson, & Moller, 2009), and persistent prosodic abnormalities are commonly seen in adults with 22q, which would suggest core speech coordination and timing difficulties (Kirschner & Baylis, 2014; Persson et al., 2017). Speakers with childhood (and adult) dysarthria, idiopathic language impairment, and CAS have been reported to have potential deficits in spatiotemporal precision (Cantiniaux et al., 2010; Goffman, 1999; Shriberg, Lohmeier, Strand, & Jakielski, 2012). Two findings for speech motor delay in studies to date are also of interest for research directions on MSDs in children and adolescents with 22q.

First, associated research has estimated the prevalence of speech motor delay in a sample of 415 children aged 3–16 years recruited for research studies in idiopathic speech delay to be 12% (Shriberg et al., 2018a). This estimate is approximately half the averaged prevalence rates for speech motor delay (26.8%) described for the four groups of youth in the present article: 22q (29.4%), DS (26.7%), FXS (28.6%), and GAL (22.6%). Considering the genetic diversity among the four neurodevelopmental disorders, this narrow range of prevalence estimates for SMD (i.e., 22.6%–29.4%) is notable. If supported in cross-validation studies with the same and different types of neurodevelopmental disorders, such prevalence findings could suggest common neuromotor consequences of diverse pathogenomic networks. That is, although the individual speech, prosody, and voice signatures of speech motor delay, childhood dysarthria, and CAS clearly arise from different deficits in speech processing (i.e., neuromotor execution deficits in speech motor delay and childhood dysarthria versus planning/programming deficits in CAS), the three MSDs also share spatiotemporal precision and stability deficits in rate, stress, and laryngeal and resonance quality (Shriberg et al., 2018a; Shriberg, Strand, Jakielski, & Mabie, 2018). A speculation first proposed in Shriberg (2017) and discussed in the two citations in the previous sentence is that false positives for CAS could be true positives for speech motor delay.

A second finding from associated retrospective longitudinal research is that speech motor delay may be the later, persistent stage of MSD in children and adults who previously met SDCS criteria for CAS, with or without concurrent childhood dysarthria (Shriberg et al., 2018a). If such findings are validated, the present lower prevalence estimates for CAS in youth with 22q in comparison with prior estimates in samples of children with 22q may be associated with differences in the ages of participants.

### Childhood Dysarthria in Speakers With 22q

Because of the widely reported prevalence of hypotonia in children and adults with 22q (e.g., Gerdes et al., 1999; McDonald-McGinn et al., 1997), a motor speech profile consistent with flaccid dysarthria was expected to be supported using the present Dysarthria Subtype Indices (see Table 2). Approximately half of the participants with 22q had a history of swallowing difficulties, which tend to be more frequent in children with dysarthric features than in children with apraxic features. The few present findings supporting three dysarthria subtypes are more consistent with the significant heterogeneity reported in neuroimaging studies of 22q, including an array of abnormalities in gray and white matter, cranial nerve function, and abnormal neural connectivity (Karayiorgou, Simon, & Gogos, 2010; Simon, Ding, et al., 2005; Villalon-Reina et al., 2013).

Interestingly, 33.3% of the participants were perceived by their parent as speaking too fast, and none was reported to use a slow rate of speech, with the latter more consistent with a flaccid and/or hypokinetic dysarthria subtype. With sufficient sample sizes of participants meeting criteria for childhood dysarthria, research in 22q could be informative for current research supporting associations between 22q and Parkinson's disease (Booij, van Amelsvoort, & Boot, 2010; Butcher et al., 2013; Zaleski et al., 2009). Specifically, recent studies that link 22q with hypokinetic dysarthria (e.g., Persson et al., 2017) have implications for the possibility of early markers of dopaminergic disease in children and adolescents. Future research should explore the potential link between dopamine dysregulation and associated speech and motor speech deficits in children with 22q. Further collaboration between speech and neuropsychiatric researchers may also be useful to further define the speech phenotype across the life span in persons with 22q. Last, application of measures sensitive to progression of speech deficits, used in other populations with known MSDs (Sussman & Tjaden, 2012), could be applied to further characterize risk or lead to an earlier identification of neurologic sequelae of this syndrome.

### CAS in Children With 22q

In a study that used the same methods as those used in this study, the prevalence of CAS in children recruited for research in idiopathic speech delay was estimated at 2.4% (Shriberg et al., 2018a). The present prevalence estimate of CAS in participants with 22q, approximately 23.6% (including childhood dysarthria and CAS), is substantially higher than a population estimate of 0.01% (Shriberg et al., 2018a) but considerably lower than the 36%–54% prevalence of CAS in speakers with 22q reported in Mills et al. (2006) and Kummer et al. (2007). As noted, overdiagnosis of CAS in idiopathic speech delay and in children with neurodevelopmental disorders has been reported worldwide. Methodological differences in diagnostic criteria across studies using a variety of perceptual and acoustic signs of CAS are one likely source of differences in prevalence estimates of CAS in children and youth with speech sound disorders. As cited previously, a speculation is that speech motor delay could account for excessive false positives for CAS. In the present context, speech findings for children with 22q add additional variables that may contribute to the percept of CAS, including unusual speech errors associated with conductive hearing loss, compensatory misarticulations including pervasive glottal stop substitutions, hypernasality, and expressive language impairment underlying poor speech intelligibility, especially at early ages. Such deficits may lead to the presumption of CAS in younger children with 22q before an accurate differential diagnosis of VPD, which may not occur until the age of 5–6 years or later (e.g., Filip et al., 2013; Spruijt, ReijmanHinze, Hens, Vander Poorten, & van der Molen, 2012).

Interestingly, in comparison with the present motor speech classification prevalence findings for CAS (23.6%; CAS plus childhood dysarthria and CAS, see Figure 1), parents of 30% of the participants with 22q in this study reported that their child had a current or prior diagnosis of CAS (*n* = 4) or childhood dysarthria (*n* = 1). This also suggests the possibility that several children with 22q were either misdiagnosed with CAS at younger ages and/or that the diagnosis of childhood dysarthria was missed in the clinical setting, potentially due to the focus on other specific articulation and/or resonance disorders in this population.

### Speech Disorders, MSDs, and Cognitive-Language Proficiency

Sample size limitations precluded regression analyses to explore associations of cognitive–linguistic status with speech and motor speech status in children with 22q. Past studies of younger children with 22q have offered conflicting findings, with some studies consistent with links between cognitive-language domains and articulation (Baylis et al., 2008) and others suggesting a dissociation between domains (Scherer et al., 1999). Findings from this study do not clearly converge on either of these perspectives. Relative to speech disorders, articulation proficiency (based on GFTA-2 standard scores) was highly variable in the group with 22q. In some children with 22q, articulation was generally commensurate with cognitive and language status scores (e.g., Participant 17; see Table 5); however, in other cases, articulation proficiency was significantly better (e.g., Participant 8) or worse (e.g., Participant 4) than cognitive–linguistic performance may suggest. Participants in two of the comparison groups (DS and FXS), however, had lower IQ and language scores than children with 22q, but similar or higher speech proficiency.

### MSDs in Speakers With 22q and VPD

The finding that 82.4% of the participants with 22q had current and/or a history of VPD symptoms is consistent with the 22q literature (Kirschner & Baylis, 2014). Only three of the 17 (17.6%) children with 22q had no current or past symptoms of VPD. Nine of the 17 participants (52.9%) had minimal to no current signs of VPD. As reviewed, the high prevalence of MSDs in the present cohort with 22q was not associated with VPD signs or severity historically or at assessment. Future studies in younger children, before surgical management, would allow for a better understanding of the potential associations between MSD, baseline speech resonance, and other features of VPD.

## Conclusion

### Study Limitations

A primary methodological limitation of this study is the small sample size of participants with 22q, with possible consequences for sample representativeness and interpretations of and generalizations from the statistical data. This constraint is not unique to this study, with the few prospective published studies of speech disorders in speakers with 22q, including sample sizes of 4–21 participants. The present findings are viewed as initial estimates of the prevalence of MSDs in youth with 22q.

Another potential representativeness constraint is ascertainment bias, which is also pervasive in the 22q literature. Participants in families who elected to participate in this study in response to recruitment materials may overrepresent speakers with more severe speech difficulties because such families may have been most motivated to gain new information or a better understanding of their child's speech condition. A major consequence of the sample size is that it prohibited group-wise descriptive and inferential statistical analyses of potentially informative speech, prosody, and voice findings for participants in each of the four MSD classifications.

A third study limitation, noted in relation to the possibility of normalization of CAS in older children, is the advanced age of participants (*M* = 10.3 years, *SD* = 3.3 years, range = 5–18 years) relative to VPD management. Ten (58.8%) of the participants had undergone surgical VPD management before their participation in this study, and one participant was wearing a palatal lift prosthesis at assessment. Eight of the 10 (80%) children in this study who had undergone prior VPD surgical management had at least mild signs of VPD, which is also consistent with the 22q literature (Spruijt et al., 2012). A goal of VPD surgery is to normalize or mitigate hypernasality, nasal emission, and pressure-associated deficits in consonant production. It is not possible to estimate the presurgical severity of VPD, just as it cannot be predicted how speech therapy may have impacted the speech disorder classification findings for participants with 22q and in the three comparative groups. As shown in Table 6, only one participant was rated as having severe VPD symptoms, seven were rated as having rated mild to moderate, and nine participants were rated as having minimal to no signs of VPD.

Last, although the primary rater of VPD symptoms (the first author) did not know the participants' SDCS motor speech classifications at the time the perceptual ratings of speech were made, future studies should include ratings of speech by judges with no knowledge of diagnosis or surgical status. In this study, which yielded high levels of agreement between the two raters, only the second rater had no information about the participants.

## Conclusion

This report appears to be the first prospective study using standardized measures to estimate the prevalence of both speech disorders and MSDs in the same children and adolescents with 22q, to compare those prevalence findings with prevalence findings obtained in studies of three other types of complex neurodevelopmental disorders, and to assess associations between motor speech findings in participants with 22q and VPD history and status. Cross-validation studies of the prevalence estimates and associated findings would appear to be warranted in studies that include larger and more diverse samples of persons with 22q and neuroimaging and craniofacial speech assessment modalities.

## Figures and Tables

**Figure B1.**
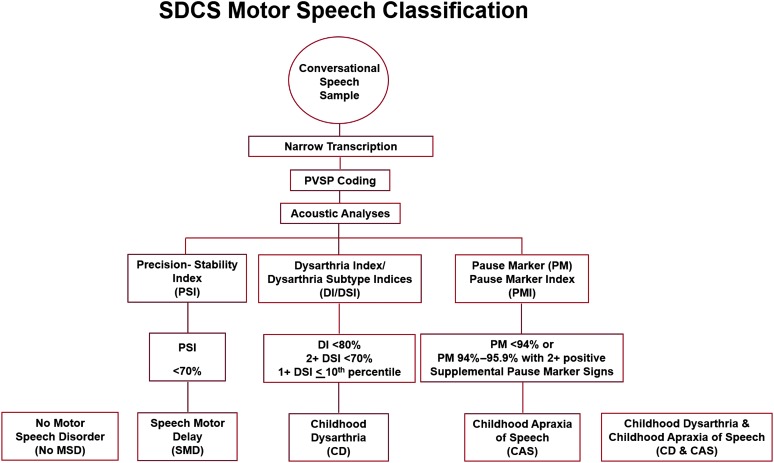
Summary of the procedure used by the Speech Disorders Classification System (SDCS) to classify a speaker's motor speech status. PVSP = Prosody-Voice Screening Profile.

## Appendix A

### Abbreviations

**Table:**

**Units and symbols**	
dB	decibel
ms	milliseconds
*n*	count
%	percentage
**Measures and classifications**	
CAS	Childhood apraxia of speech
CD	Childhood dysarthria
DS	Down syndrome
DI	Dysarthria Index
DSI	Dysarthria Subtype Indices
MSD	Motor speech disorder
NSA	Normal(ized) speech acquisition
PM	Pause Marker
PMI	Pause Marker Index
PVC	Percentage of vowels correct
PSD	Persistent speech delay
PSE	Persistent speech errors
PSI	Precision–Stability Index
PVSP	Prosody-Voice Screening Profile
SD	Speech delay
SDCS	Speech Disorders Classification System
SDCSS	Speech Disorders Classification System Summary
SE	Speech errors
SMD	Speech motor delay
SPMS	Supplementary Pause Marker signs

## Appendix B

### Motor Speech Classification: Procedures and Measures

This Appendix provides information on the procedure and measures the Speech Disorders Classification System (SDCS) uses to classify a speaker's motor speech status. Figure B1 is a summary of the procedure. Additional information on methods is described in the following sections, in the research articles and technical reports cited in the text, and in Programs to Examine Phonetic and Phonologic Evaluation Records (PEPPER, 2019).

As shown in Figure B1, the procedure begins with an audio-recorded conversational speech sample, obtained following the guidelines described in PEPPER (2019). Next, narrow phonetic transcription, prosody-voice coding, and acoustic analyses are completed following PEPPER guidelines and entered into the software. Third, the software computes scores on the three measures, as described in the following sections. Last, using the rules for each of the three measures shown in Figure B1 and in the following sections, the computer program classifies the speaker's motor speech status into one of five classifications. The following sections include descriptions of each measure, including copies of the signs and information on the calculations for each sign in two of the measures.

#### The Precision–Stability Index

Tables B1 and B2 include the 13 perceptual and 19 acoustic signs of precision and stability of speech that comprise the 32-item Precision–Stability Index (PSI; Shriberg et al., 2010a). Table B1 is a facsimile of the PSI scoring sheet, and Table B2 includes information on the calculations completed by the PEPPER (2019) software for each sign. The PSI was developed as a measure to identify and quantify the SDCS classification of motor speech disorders termed *speech motor delay*. The 32 signs are divided into three domains of speech and seven domains of prosody and voice. Tables B1 and B2 are the PSI forms for individual speakers; outputs are also available that summarize findings from a group of speakers. A technical report includes additional psychometric information and empirical findings for this measure (Mabie & Shriberg, 2017).

As shown in Figure B1, the data for each of the signs are obtained from a standardized procedure to obtain a sample of continuous or conversational speech, including procedures useful for children with low verbal output (McSweeny, 1998; Shriberg & Kwiatkowski, 1985). Phonetic transcription, prosody-voice coding, and acoustic analyses procedures are described and referenced in Shriberg et al. (2010a), with additional information in PEPPER (2019). A database of 150 typical speakers used to standardize speech, prosody, and voice signs of speakers 3–17 years of age includes five speakers of the same sex at each age (Potter et al., 2012). A reference database of 50 speakers used to standardize signs for 20- to 80-year-old speakers includes four speakers of the same sex for speakers across each decade from 20 to 69 years of age and five speakers of the same sex from 70 to 79 years of age (Scheer-Cohen et al., 2013).

A *z* score cutoff of 1.25 is used to divide performance on each sign as within or not within the typical range. Directionality of the *z* score changes for some signs; for most signs, a minus *z* score indicates lower precision or lower stability. *z* scores equal to or less than 1.24 are considered “not positive” for the sign and coded “0.” *z* scores greater than 1.25 are considered “positive” for the sign and coded “1.” The PSI percentage score is calculated by dividing the number of positive signs by 32 (or less if missing data) and subtracting the quotient from 100, so that lower percentage scores reflect reduced precision and stability. As shown in Figure B1, to be classified as PSI+ (i.e., SMD), a speaker's PSI score is required to be less than 70%.

#### The Dysarthria Index and Dysarthria Subtype Indices

Tables B3 and B4 include the 19 perceptual and 15 acoustic signs that comprise the Dysarthria Index and Dysarthria Subtype Indices (DI/DSI). Information for each of the signs is obtained from the same sample of continuous or conversational speech as used for the PSI and processed using the same transcription, prosody-voice, and acoustic methods and reference databases to standardize scores. Table B3 is a facsimile of the DI/DSI form for individual speakers; outputs are also available that summarize findings from a group of speakers. Table B4 includes information on the calculations completed by the PEPPER (2019) software for each sign. The DI/DSI were developed as measures to identify and quantify the SDCS classifications of motor speech disorders termed *childhood dysarthria* (CD) and *CD and childhood apraxia of speech*.

The 34 items that operationalize and quantify signs of dysarthria in the DI/DSI were based on the adult-onset signs of dysarthria, including the weightings of each sign (“1” or “2,” bolded) for subtypes of dysarthria, as described in Duffy (2013). A *z* score cutoff of 1.50 *SD* units is used to divide performance on each sign as within or not within the typical range. The same procedures used for the PSI scores are used to code each of the 34 DI/DSI items. Also, as described in Mabie and Shriberg (2017), the item weightings for each of the dysarthria subscales shown in Table B3 (which are the same weightings as those in Duffy [2013]) are used to derive percentage scores for each of the five dysarthria subscales. As shown in Figure B1, three criteria must be met to code a speaker as positive for dysarthria. First, the DI score must be less than 80.0%. Second, two or more of the five dysarthria subtypes must have percentage scores less than 70.0%. Last, at least one dysarthria subtype percentile is required to be less than or equal to the 10th percentile (see Mabie & Shriberg, 2017, for how percentile reference data were obtained). Because the same signs are used in as many as three of the five indices, some of the five DSI have high correlation coefficients with one another (Shriberg & Mabie, 2017). Until additional research is available, the descriptive percentile data are used only to support the possibility of pure or mixed subtypes of CD.

#### The Pause Marker and Pause Marker Index

The Pause Marker (PM) is a diagnostic marker to identify speakers with CAS and discriminate CAS from SMD and CD (Shriberg et al., 2017a, 2017b, 2017c, 2017d). As shown in Figure B1, speech data for the PM are obtained from the same sample of conversational speech as used for the PSI and DI/DSI and processed using the same transcription, prosody-voice, and acoustic methods and reference databases to standardize PM scores. There is no PM form to include in this Appendix. As shown in Figure B1, CAS classification requires provisions for several quantitative outcomes.

The PM score is based on the occurrence of four types of inappropriate between-words pauses collectively termed *Type I pauses*. Tilkens et al. (2017) include reference information for the four Type I pauses as well as for the four inappropriate between-words pauses (*Type II pauses*) that provide additional information on speech processes for clinical and research applications in CAS.

The PM score is the percentage (subtracted from 100%) of Type I pauses that occur in 24 utterances of a continuous speech sample that meet eligibility criteria for the Prosody-Voice Screening Profile (Shriberg, Kwiatkowski, & Rasmussen, 1990). A minimum of 40 between-words pause opportunities must occur in the speech sample in order to obtain a valid PM score. PM scores lacking 40 between-words pauses are classified as indeterminate. A PM score above 96% (PM−) is classified as within the typical range and the speaker is classified as negative for CAS (CAS−). A PM score below 94% (PM+) is classified as not within the typical range and the speaker is classified as positive for CAS (CAS+).

A PM score from 94% to 95.9% is termed a *marginal* PM score. To resolve the CAS classification of a speaker with a marginal PM score, findings from three signs termed the *supplemental PM signs* are used. CAS+ classification of a marginal PM score requires that at least two of the three supplemental PM signs (slow rate, inappropriate stress, transcoding errors) are positive. Marginal PM scores that cannot be resolved by SPMS findings (due to missing data or insufficient pause opportunities) are also classified as indeterminate.

To scale the severity of CAS for clinical and research needs, the Pause Marker Index divides PM+ scores into four ordinal levels (Shriberg et al., 2017d). “Mild” CAS severity scores include PM scores from 90.0% to 93.9%, “mild–moderate” severity scores include PM scores between 85.0% and 89.9%, “moderate–severe” severity scores include PM scores between 80.0% and 84.9%, and “severe” severity scores include PM percentages below 80.0%.

**Table B1.** Precision–Stability Index (PSI).

PSI: Individual							
Linguistic domain	Sign				Sign values		
	No.	Description	Assessment mode^a^		Value	*z* Score^b^	Code^c^
Vowels			P	A			
	1	Reduced dispersion of corner vowels from center		X			
	2	Reduced dispersion of corner vowels from ^		X			
	3	Reduced average pairwise distance of corner vowels		X			
	4	Increased duration of corner vowels		X			
	5	Increased duration for middle vowels and diphthongs		X			
	6	Reduced % vowel phoneme target consistency	X				
	7	Reduced % vowel target consistency	X				
Consonants							
	8	Reduced % correct glides	X				
	9	Increased relative distortion index: sibilants	X				
	10	Reduced % dentalized sibilants of distorted sibilants	X				
	11	Increased relative distortion index for early consonants	X				
	12	Decreased first moment on /s/ initial singletons		X			
	13	Increased sqrt of the second moment for /s/ initial singletons		X			
	14	Increased sqrt of the second moment for /s/ initial, and /s/ and /z/ final singletons		X			
	15	Increased all consonant–consonant duration		X			
Vowels and consonants							
	16	Increased Diacritic Modification Index (DMI) class: place %	X				
	17	Increased DMI class: duration %	X				
	18	Increased % of epenthesis errors	X				
Phrasing							
	19	Increased PM errors: % of addition, breath, repeat, or long	X				
Rate							
	20	Reduced syllables per second (without pauses)		X			
	21	Increased syllable length in ms (without pauses)		X			
Stress							
	22	Increased % of prosody-voice (PV) 15/16 EE (excessive/equal stress) codes of all coded utterances without fast/acceleration (uncircled and circled)	X				
	23	Increased % of PV15/16 EE codes of all PV15/16 codes (uncircled and circled)	X				
Loudness							
	24	Decreased intensity difference, dB fricative + vowel		X			
Pitch							
	25	Decreased F0 for all vowels and diphthongs		X			
	26	Decreased range of characteristic F0 for delimited vowels/diphthongs		X			
Laryngeal quality							
	27	Increased % jitter for vowels^b^		X			
	28	Increased % shimmer for vowels^b^		X			
	29	Decreased HNR dB for vowels		X			
Resonance quality							
	30	Increased % voice quality resonance wrong	X				
	31	Decreased F1 /a/ (nasal)		X			
	32	Decreased F2 for high vowels (nasopharyngeal)		X			
No. of positive signs							
No. of signs with data							
Average sign *z* score							
Signs score							

*Note.* PM = Pause Marker. Sqrt = square root; HNR = harmonics-to-noise ratio.

a A = acoustic; P = perceptual.

b *z* scores referenced to age–sex matched typically developing speakers (Potter et al., 2012; Scheer-Cohen et al., 2013).

c Code: 0 = *not positive on variable*; 1 = *positive on variable* (*z* score ≤ 1.25). *z* scores reversed for increased.

**Table B2.** Precision–Stability Index Sign definitions.

No.	Sign	Mode^a^		Calculation
		P	A	
1	Reduced dispersion of corner vowels from center		X	There are four corner vowels. The center is defined using the average first and second formant frequencies over the four corner vowels. Dispersion is the weighted mean of the corner vowels of the distance to that center. “Weighted” means each vowel occurrence is separately included in the dispersion calculation. In comparison, the center location calculation is unweighted. The average formant frequency pairs are separately calculated for each of the four vowels. The resulting four frequency pairs are then averaged to get the vowel center.
2	Reduced dispersion of corner vowels from ^		X	The location of any vowel is the average first and second formant frequencies. Dispersion is the average distance of the location of each of the four corner vowels to the location of four.
3	Reduced average pairwise distance of corner vowels		X	This is the average distance from the location of each corner vowel to the location of the other three corner vowels.
4	Increased duration of corner vowels		X	The weighted mean of the length of the corner vowels. “Weighted” means each vowel occurrence is separately included in the calculation.
5	Increased duration for middle vowels and diphthongs		X	This includes eight monophthongs and five diphthongs.
6	Reduced % vowel phoneme target consistency	X		A type is a distinct Y-line word considering just the phonemes (see PEPPER [2019]for a description of X, Y, and Z lines). A token is a specific occurrence. A type can have many tokens. Tokens that have anything questionable in the X or Y lines are ignored. Phonemes that are questionable in the Z line are ignored. Cases where a phoneme occurs twice (e.g., “b” in “bob”) count as two types. For a given phoneme in a type, consistency looks at just the corresponding position in the tokens for errors. Within a type, consistency is defined as the probability, for the selected phoneme, that any two randomly selected (without replacement) tokens have the same obtained result (considering either phoneme only or phoneme and diacritics). For error consistency, the two tokens are selected just from those with errors; for target consistency, they are selected from all the tokens (assuming at least one token has an error). For example, if three different obtained results occur with frequencies of I, J, and K, then this probability is: $\frac{I*\left(I-1\right)+J*\left(J-1\right)+K*\left(K-1\right)}{N*\left(N-1\right)}$ where *N* = *I* + *J* + *K*. To combine these probabilities over types, we weight each probability by *N* − 1, because a type with only one eligible token gives us no information. For our “numerator,” we store the sum of each type's probability times its *N* − 1. For our “denominator,” we store the sum of each type's *N* − 1. Target consistency considers only those types with at least two tokens where at least one has an error. Phoneme consistency considers just substitutions and deletions to be errors.
7	Reduced % vowel target consistency	X		Complete consistency considers substitutions, deletions, and distortions to be errors. Distortions are diacritics on the Z line only (produced but not intended) aside from stress and juncture diacritics.
8	Reduced % correct glides	X		There are two English glides.
9	Increased relative distortion index: sibilants	X		Percentage of sibilant distortions of all sibilant errors (distortions, substitutions, and deletions).
10	Reduced % dentalized sibilants of distorted sibilants	X		Distortions are diacritics on the Z line only (produced but not intended) aside from stress and juncture diacritics. There are three English sibilants.
11	Increased relative distortion index for early consonants	X		Percentage of distorted early eight consonants of all early eight errors.
12	Decreased first moment on /s/ initial singletons		X	See centroid definition at https://en.wikipedia.org/wiki/Spectral_centroid
13	Increased sqrt of the second moment for /s/ initial singletons		X	Sqrt is the abbreviation for square root.
14	Increased sqrt of the second moment for /s/ initial and /s/ and /z/ final singletons		X	The same as the previous item except that final /s, z/ are included.
15	Increased all consonant–consonant duration		X	Average length in milliseconds of all consonant pairs where the consonants are less than 0.1 s apart and they are not the same consonant or a cognate.
16	Increased Diacritic Modification Index (DMI) class: place %	X		Percentage of phonemes with one or more tongue configuration or position diacritics.
17	Increased DMI class: duration %	X		Percentage of phonemes lengthened or shortened.
18	Increased % of epenthesis errors	X		Percent of epenthesis errors (vowel addition) by token (by word).
19	Increased PM errors: % of addition, breath, repeat, or long	X		Percentage of pause opportunities with one or more of addition, breath, repeat, or long. (Counted even if grope, change, or abrupt is also present.)
20	Reduced syllables per second (without pauses)		X	Syllables per second for first 12 coded utterances after pauses are removed.
21	Increased syllable length in ms (without pauses)		X	Uses the first 12 coded utterances.
22	Increased % of prosody-voice (PV) codes 15/16 EE codes of all coded utterances without fast/acceleration (uncircled and circled)	X		EE is the abbreviation for excessive/equal stress, an inappropriate stress pattern that can occur on utterances that have PVSP inappropriate code of 15 or 16 (see pp. 31–32 of the PVSP manual [Shriberg, Kwiatkowski, & Rasmussen, 1990]). Inappropriate fast and/or accelerated speech (PV11/12) is defined as greater than four syllables per second for children and greater than six syllables per second for adolescents and adults. Uncircled and circled are treated as inappropriate and appropriate, respectively. Circled codes give speakers the benefit of the doubt when a coding decision is difficult to make.
23	Increased % of prosody-voice codes 15/16 EE codes of all PV15/16 codes (uncircled and circled)	X		The same as above except the denominator is the number of PV15/16 codes of any kind.
24	Decreased intensity difference, dB, fricative + vowel		X	For a fricative–vowel pair, the intensity difference is the intensity of the vowel in dB minus the intensity of the fricative in dB. This uses the average intensity difference over all fricative–vowel pairs in the transcript where both phonemes have been delimited during the acoustic analysis.
25	Decreased F0 for all vowels and diphthongs		X	F0 is the fundamental frequency at the characteristic point for those vowels and diphthongs that were delimited during the acoustic analysis.
26	Decreased range of characteristic F0 for delimited vowels/diphthongs		X	This is the overall maximum F0 minus the overall minimum F0.
27	Increased % jitter for vowels^b^		X	“Jitter is the cycle-to-cycle variation of fundamental frequency, i.e., the average absolute difference between consecutive periods.”
28	Increased % shimmer for vowels^b^		X	“Shimmer (dB) is expressed as the variability of the peak-to-peak amplitude in decibels, i.e., the average absolute base-10 logarithm of the difference between the amplitudes of consecutive periods, multiplied by 20.”
29	Decreased HNR dB for vowels		X	TF32 (Milenkovic, 2001 ^c^) calculates the SNR (signal-to-noise ratio) in dB. To calculate the HNR (harmonics-to-noise ratio): Power = 10 to the (SNR/10) power HNR = 10 * log10(Power − 1)
30	Increased % voice quality resonance wrong	X		Inappropriate resonance in the PVSP includes inappropriate codes 30, 31, and 32 (nasal, denasal, and nasopharyngeal)
31	Decreased F1 /a/ (nasal)		X	First formant frequency for /a/
32	Decreased F2 for high vowels (nasopharyngeal)		X	Second formant frequency for /i/ and /u/

*Note.* PVSP = Prosody-Voice Screening Profile; PM = Pause Marker.

a A = acoustic; P = perceptual.

b Jitter and shimmer definitions adapted from “Jitter and Shimmer Measurements for Speaker Recognition,” by M. Farrús, J. Hernando, and P. Ejarque, 2007, *Proceedings of the Interspeech*, 778–781.

c *TF32*: Department of Electrical and Computer Engineering, University of Wisconsin-Madison.

**Table B3.** Dysarthria Index (DI) and the five Dysarthria Subtype Indices (DSI).

DI and DSI: Individual											
Linguistic domain	Sign no.	Description	Assessment mode^a^		DI sign values		Five DSI^b^				
			P	A	*z* Score^c^	Code^d^	Ataxia	Spastic	Hyperkinetic	Hypokinetic	Flaccid
Vowels											
	1	Increased percentage of vowels/diphthongs distortions	X				**X (2)**		**X (2)**		
Consonants											
	2	Number of nasal emissions	X								**X (2)**
	3	Increased percentage of weak consonants	X								X (1)
Vowels and consonants											
	4	Increased Diacritic Modification Index class duration	X				X (1)		X (1)		
Phrasing											
	5	Increased slow/pause time	X						X (1)	**X (2)**	
Rate											
	6	Increased slow articulation/pause time	X				X (1)	**X (2)**	X (1)		
	7	Decreased average syllable speaking rate (with pauses)		X			X (1)	**X (2)**	X (1)		
	8	Decreased average syllable articulation rate (without pauses)		X			X (1)	**X (2)**	X (1)		
	9	Increased fast rate	X							**X (2)**	
	10	Decreased stability of syllable speaking rate		X					X (1)	**X (2)**	
Stress											
	11	Increased excessive/equal/misplaced stress	X				**X (2)**	X (1)			
	12	Increased reduced/equal stress	X							**X (2)**	
Loudness											
	13	Decreased stability of Speech Intensity Index		X			**X (2)**		**X (2)**		
	14	Increased stability of Speech Intensity Index		X				X (1)		**X (2)**	X (1)
	15	Increased soft	X							**X (2)**	X (1)
	16	Decreased Speech Intensity Index		X						**X (2)**	X (1)
Pitch											
	17	Increased low pitch/glottal fry	X					**X (2)**	X (1)		
	18	Increased low pitch	X					**X (2)**	X (1)		
	19	Decreased F0 for all vowels and diphthongs		X				**X (2)**	X (1)		
	20	Decreased range of char. F0 among vowels		X				X (1)	X (1)	**X (2)**	X (1)
	21	Decreased stability of F0 for all vowels and diphthongs		X			X (1)				
Laryngeal quality											
	22	Increased breathy	X							X (1)	**X (2)**
	23	Increased rough	X					X (1)	X (1)		
	24	Increased strained	X					X (1)	X (1)		
	25	Number of utterances with [TREM] (tremulous) comment	X						X (1)		
	26	Increased break/shift/tremulous	X					**X (2)**	X (1)		
	27	Increased multiple features	X					**X (2)**	**X (2)**		
	28	Number of diplophonia	X								**X (2)**
	29	Increased % jitter for vowels		X			X (1)				
	30	Decreased stability of jitter for vowels		X			X (1)				
	31	Increased % shimmer for vowels		X			X (1)				
	32	Decreased stability of shimmer for vowels		X			X (1)				
Resonance quality											
	33	Increased nasal	X					X (1)	X (1)	X (1)	**X (2)**
	34	Decreased F1 for /a/ (nasal)		X				X (1)	X (1)	X (1)	**X (2)**
		Unweighted total possible points					12	15	19	11	10
		Weighted total possible points					15	23	22	19	15
**DI summary**			**DSI summary**								
**No. of positive signs**							**Ataxia**	**Spastic**	**Hyperkinetic**	**Hypokinetic**	**Flaccid**
No. of signs with data			Percentage of positive signs								
Average sign *z* score			DSI (% nonpositive weighted)								
DI (% nonpositive signs)			DSI percentile score								

a A = Acoustic; P = perceptual.

b Very frequent: 80.0%–100%; frequent: 60.0%–79.9%; somewhat frequent: 40.0%–59.9%; somewhat infrequent: 20.0%–39.9%; infrequent: 0.0%–19.9%.

c *z* scores referenced to age–sex matched, typically developing speakers (Potter et al., 2012; Scheer-Cohen et al., 2013). For the three “Number of” items (2, 25, and 28), this column has a count rather than a *z* score.

d Code: 0 = *not positive on variable*; 1 = *positive on variable* (*z* score ≤ 1.50 or “Number of” ≥ 2).

**Table B4.** Dysarthria Index and the five Dysarthria Subtype Indices' sign definitions.

No.	Sign	Mode^a^		Calculation
		P	A	
1	Increased percentage of vowel/diphthong distortions	X		Distortions are diacritics on the Z line only (produced but not intended) aside from stress and juncture diacritics (see PEPPER [2019] for a description of X, Y, and Z lines).
2	Number of nasal emissions	X		Note that this is a count rather than a percentage. Two or more is coded as significant.
3	Increased percentage of weak consonants	X		The “check” diacritic. The “check” diacritic is used to indicate a weakly produced consonant.
4	Increased diacritic modification index class duration	X		The lengthened diacritic (:) or shortened diacritic (>).
5	Increased slow/pause time	X		Rate of less than two syllables per second due to long pause time only (prosody rate code 10).
6	Increased slow articulation/pause time	X		Rate of less than two syllables per second due to slow articulation and pause time (prosody rate code 9).
7	Decreased average syllable speaking rate (with pauses)		X	The average over the first 12 coded utterances given in the Prosody-Voice Screening Profile (PVSP) log of the number of syllables divided by the duration of the utterance as given in the acoustic analysis.
8	Decreased average syllable articulation rate (without pauses)		X	The average over the first 12 coded utterances of the number of syllables divided by the duration of the utterances, less the pause time.
9	Increased fast rate	X		Rate greater than four syllables per second for children or greater than six syllables per second for adolescents/adults (prosody rate code 11).
10	Decreased stability of syllable speaking rate		X	For any measure that occurs multiple times within a source, the stability of that measure can be calculated: Stability = 100*(1-StanDev/Mean) where “*” indicates multiplication, “/” indicates division, and “StanDev” is the standard deviation. If an item is completely stable, StanDev is zero and stability is 100. As StanDev increases as a fraction of the mean, the stability value drops.
11	Increased excessive/equal/misplaced stress	X		Inappropriate stress that is excessive/equal or misplaced (prosody stress code 15; see pp. 31–32 of the PVSP manual [Shriberg, Kwiatkowski, & Rasmussen, 1990]).
12	Increased reduced/equal stress	X		Inappropriate reduction of stress in stressed syllables, plus lack of appropriate stress variation (prosody stress code 14).
13	Decreased stability of Speech Intensity Index (SII)		X	The SII quantifies the difference in dB between a stop or fricative and the following vowel.
14	Increased stability of SII		X	The SII quantifies the difference in dB between a stop or fricative and the following vowel.
15	Increased soft	X		Inappropriate soft voice; judged to be socially unacceptable in face-to-face communication (voice loudness code 17).
16	Decreased SII		X	See Items 13 and 14 for SII definition relative to the stability of SII.
17	Increased low pitch/glottal fry	X		Inappropriate low-pitched, periodic “popping” voice quality distributed across an utterance (voice pitch code 19).
18	Increased low pitch	X		Pitch is inappropriately low for the speaker's age or gender (voice pitch code 20).
19	Decreased F0 for all vowels and diphthongs		X	F0 is the fundamental frequency at the characteristic point for vowels and diphthongs delimited in the acoustic analysis.
20	Decreased range of char. F0 among vowels		X	This is the overall maximum F0 minus the overall minimum F0.
21	Decreased stability of F0 for all vowels and diphthongs		X	See Item 10 for the definition of stability.
22	Increased breathy	X		Inappropriate laryngeal quality with insufficient vocal tone relative to unvoiced airflow (voice laryngeal code 23).
23	Increased rough	X		Inappropriate laryngeal quality with an aperiodic “gravelly” sound (voice laryngeal code 24).
24	Increased strained	X		Inappropriate laryngeal quality with a strident, tense sounding vocal tone (voice laryngeal code 25).
25	Number of utterances with [TREM] (tremulous) comment	X		Note that this is a count rather than a percentage. Two or more is coded as significant.
26	Increased break/shift/tremulous	X		Occurrence of a voice break, a pitch shift, and/or a tremulous vowel (voice laryngeal code 26).
27	Increased multiple features	X		Inappropriate co-occurring laryngeal features not covered under one laryngeal code (voice laryngeal code 29).
28	Number of diplophonia	X		Note that this is a count rather than a percentage. Two or more is significant (voice laryngeal code 28).
29	Increased % jitter for vowels^b^		X	“Jitter is the cycle-to-cycle variation of fundamental frequency, i.e., the average absolute difference between consecutive periods.”
30	Decreased stability of jitter for vowels		X	See Item 10 for the definition of stability.
31	Increased % shimmer for vowels^b^		X	“Shimmer (dB) is expressed as the variability of the peak-to-peak amplitude in decibels, i.e., the average absolute base-10 logarithm of the difference between the amplitudes of consecutive periods, multiplied by 20.”
32	Decreased stability of shimmer for vowels		X	See Item 10 for the definition of stability.
33	Increased nasal	X		Inappropriate excess nasality in assimilative and/or assimilative nasality contexts (voice resonance code 30).
34	Decreased F1 for /a/ (nasal)		X	First formant frequency for /a/.

a A = acoustic; P = perceptual.

b Jitter and shimmer definitions adapted from “Jitter and Shimmer Measurements for Speaker Recognition,” by M. Farrús, J. Hernando, and P. Ejarque, 2007, *Proceedings of the Interspeech*, 778–781.
